# Supplementary material for: Therapeutic hypothermia for neonatal hypoxic ischaemic encephalopathy in Sub-Saharan Africa: A scoping review
Source: PLoS One. 2025 Feb 6;20(2):e0315100. doi: 10.1371/journal.pone.0315100 (PMC11801734; doi:10.1371/journal.pone.0315100)
Supplement: S1 File — (PDF) [file pone.0315100.s002.pdf]

Supplementary File 1: Final Data Table

**Reviewer initials or consensus:**	
**Study demographics**	
Full citation with first 3 authors.	
Year (s) when data collected (not publication)	
Country where study conducted	
City where study conducted	
Name of facility	
Type of facility	
Level of care where cooling provided	
Type of study	
Primary Aim	
Other Objectives	
Population (participants if survey)	
Sample Size	
Proportion of cooled neonates (TH uptake if survey)	
Survey method if survey	
**Criteria for hypoxia, HIE and TH**	
Birth criteria for acute peripartum hypoxia	
Clinical Neurological Assessment Methods	
aEEG/EEG – use and classification	
Clinical criteria for Moderate-Severe HIE	
Inclusion Criteria for TH	
Exclusion Criteria for TH	
**Neonatal characteristics at presentation - babies treated with TH**	
Maternal HIV infection; (n/N) %	
Intrapartum complications/events; (n/N) %	
Chorio-amnionitis; (n/N) %	
PROM; (n/N) %	
Inborn; (n/N) %	
Male; (n/N) %	
Gestational Age (weeks)	
Birth Weight (grams)	
Birth Weight < 10th Centile; (n/N) %	
Apgar Score 1 minute	
Apgar Score 5 minute	
Proportion with early blood gas (cord/1st hour)	
pH 1st 60 minutes	
Base Deficit 1st 60 minutes	
Worst Encephalopathy Grade/Score in first 6 hours/before TH	
Worst precooling/6hrs aEEG	
Other early aEEG grade/score	
**TH procedure and complications**	
TH method (as much detail as provided)	
Age started	
Duration	
Body temperature measurement / site	
Target temperature / site	

Time to target temperature from TH start	
Rewarming method	
Rewarming duration	
TH stopped early for any reason ?	
Complications directly attributed to TH	
Other neuroprotective treatments	
ASD protocol	
Analgesia/sedation during TH	
Sodium Bicarbonate for metabolic acidosis	
Fluid & nutrition Protocol	
Special Monitoring	
Cranial Ultrasound Scan done?	
MRI available and done?	
Empiric antibiotics for all babies with HIE?	
Mechanical ventilation/NICU available for all? (If not, who excluded?)	
**Morbidity and outcomes in cooled babies**	
Proportion IIPPV; n/N (%)	
Major Morbidity:<br><br>(Inotropes/Infection/NEC/ Hypo/per glycaemia/Bleeding/PPHN)	
Worst Encephalopathy grade	
Severely abnormal 48-hour aEEG or death	
Other aEEG outcomes	
Other clinical neurological outcomes by discharge	
Hospital stay until discharge	
Time to full cup/breast feeding	
Cranial ultrasound abnormalities	
Outcome predictors by discharge/death	
Mortality	
MRI outcomes	
Clinical neurological outcomes after discharge – when and how measured	
Number lost to clinical follow-up after discharge	
Survived and normal/mildly abnormal at 12 months	
Died or CP at 12 months	
CP in survivors at 12 months	
Quality of life outcomes	
TH standard care during study? (TH opinion if survey)	
Other Comments	

Abbreviations: aEEG – amplitude integrated electro encephalography; ASD – anti-seizure drugs; CP - cerebral palsy; HIE – hypoxic ischaemic encephalopathy; IIPV – intubated intermittent positive pressure ventilation; MRI – magnetic resonance imaging; NICU – neonatal intensive care unit; NEC – necrotising enterocolitis; PPHN – persistent pulmonary hypertension; TH – therapeutic hypothermia.
